# An important step to translate single‐cell measurement into clinical practice: Stereoscopic cells

**DOI:** 10.1002/ctm2.70304

**Published:** 2025-04-14

**Authors:** Xiangdong Wang, Wanxin Duan, Xuanqi Liu, Jia Fan

**Affiliations:** ^1^ Department of Pulmonary and Critical Care Medicine Zhongshan Hospital, Fudan University Shanghai Medical College Shanghai China; ^2^ Shanghai Institute of Clinical Bioinformatics Shanghai China; ^3^ Fudan University Center of Clinical Bioinformatics Shanghai China; ^4^ Department of Liver Surgery and Transplantation and Key Laboratory of Carcinogenesis and Cancer Invasion Ministry of Education, Liver Cancer Institute, Zhongshan Hospital, Fudan University Shanghai China

## Abstract

30 April 2025: This article was published in error. The final version of record will be made fully available at a later date.

